# Role of Multifaceted Social Relationships on the Association of Loneliness with Depression Symptoms: A Moderated Mediation Analysis

**DOI:** 10.3390/healthcare11010124

**Published:** 2022-12-31

**Authors:** Yang Liu, Dandan Jiao, Mengjiao Yang, Mingyu Cui, Xiang Li, Zhu Zhu, Yuko Sawada, Kumi Watanabe Miura, Taeko Watanabe, Emiko Tanaka, Tokie Anme

**Affiliations:** 1 School of Comprehensive Human Science, University of Tsukuba, Tsukuba 3058577, Japan; 2 Department of Nursing, The First Affiliated Hospital, College of Clinical Medicine of Henan University of Science and Technology, Luoyang 471003, China; 3 Department of Cardiovascular Surgery, Affiliated Hospital of North Sichuan Medical College, Nanchong 637000, China; 4 Department of Physical Therapy, Morinomiya University of Medical Sciences, Osaka 5598611, Japan; 5 RIKEN Center for Advanced Intelligence Project, Tokyo 1030027, Japan; 6 Faculty of Nursing, Shukutoku University, Chiba 2608701, Japan; 7 Faculty of Nursing, Musashino University, Tokyo 1358181, Japan; 8 Faculty of Medicine, University of Tsukuba, 1-1-1 Tennodai, Tsukuba, Ibaraki 3058577, Japan

**Keywords:** depression, loneliness, social relationships

## Abstract

Strong relationship exists between loneliness and depression in older adults. However, the effect of multifaceted social relationships on the relationship between loneliness and depression has not been explored. The purpose of the current study was to find out how multifaceted social relationships affect the aforementioned processes. We investigated and evaluated the loneliness status, depression symptoms, social relationships, and demographic information of 1116 older adults aged ≥65 years living in rural Japan. The final 555 participants were included in the analysis. Statistical evidence showed a direct effect between loneliness and depression symptoms. Additionally, the mediation model found that social curiosity and participation acted as mediators between loneliness and depression symptoms. Further, independence and participation, independence, and feeling safe played a conditional moderating role in the model of loneliness–social curiosity–depression symptoms and loneliness–participation–depression symptoms, respectively. Interaction can be an individual moderator in the link between loneliness and depression symptoms without any mediator. The moderated mediation model suggests that social curiosity and participation could mediate the association between loneliness and depression symptoms. In this process, independence, participation, and feeling safe may act as moderators.

## 1. Introduction

Recently, human life expectancy rapidly increased [1] An unprecedented number of people are growing older, with a decline in fertility [2], and the resulting aging has become a global phenomenon [3]. In 2019, the World Health Organization reported that the number of people aged ≥60 was 1 billion, which will increase to 1.4 billion by 2030 [4].

Older adults often experience a decline in physical health, such as increased multimorbidity [5,6] and decreased instrumental activities of daily living [7,8]. These experiences increase their risk and vulnerability to depression; however, depression is not a normal part of aging [9,10]. According to the definition of the National Institute of Mental Health [11], depression (also called major depressive disorder or clinical depression) is a common but serious mood disorder. As the most frequent cause of emotional distress in later life [12], it affects people’s feelings, thinking, and handling of daily activities [11]. Furthermore, it has caused more ‘years lost’ to disability than any other disease [13] and greatly reduces the quality of life of older adults [12]. With the dramatic increase in the number of older adults, preventing depression in older adults is a great challenge.

The evidence indicated that the risk of depression decreases in both men and women after menopausal age [14]. Moreover, sex differences peak in adolescence, diminish, and remain stable in later adulthood [15]. Additionally, exercise stimulates a complex system and leads to higher resilience to stress-related mental disorders, preventing depression [16]. Economic status [17] and chronic diseases [18,19,20,21,22,23,24,25,26,27] are also associated with depression. To prevent depression in older adults, an important method is to lessen loneliness because loneliness is significantly correlated with depression symptoms [28]. Domènech-Abella et al. (2019) [29] reported a bidirectional longitudinal association between loneliness and a higher likelihood of major depressive disorder after 2 years in people aged over 50 years. Erzen and Çikrikci conducted a meta-analysis and concluded that loneliness is an important variable affecting depression [30]. Lee et al. (2021) demonstrated that higher loneliness scores at baseline were significantly associated with depression symptom scores over 12 years after controlling for other social experiences in people aged >50 years [31].

However, after clarifying that lessening loneliness helps to prevent depression in older adults, another question arises: are there other factors contributing to the effects of loneliness on depression? Social relationships have recently been suggested to explain this.

Social relationships are measured by the presence or absence of human relationships in the local community, and the frequency of relationships within one’s environment [32] was linked to loneliness and depression. Cacioppo and Hawkley(2009) indicated that loneliness can be spread among people and can further reduce their social relationships [33]. Prince et al. (1997) reported that social support deficits are strongly associated with depression [34], and Lee et al. (2020) showed that support from friends and romantic partners might lessen depression [35]. Furthermore, Arslantaş et al. (2015) reported that a lack of hobbies increases loneliness and is associated with depression symptoms [36]. Seabrook et al. (2016) and Yang et al. (2021) found that positive interactions, social support, and social connections on social networking sites lessen depression and anxiety [37,38]. To better understand the role that social relationships play in the association between loneliness and depression, social relationships are studied as a mediator and moderator. Park et al. (2019) used family and friend networks and perceived community support to assess the mediating effects of social engagement on loneliness and depression. [39] Burholt and Scharf (2014) focused on how social resources (marital status, sociability, religious and other community groups) and social participation (number of activities attended in a month) mediate loneliness and depression [40]. In addition, Liu et al. (2016) measured the mediating effect of social support on loneliness and depression in 320 older adults using the support of family, friends, and others. [41]. Zhao et al. (2018) used the same strategy as Liu et al. (2016) to explore the moderating effect of social support on loneliness and depression [42]. However, social relationships were measured using multifaceted factors, and each aspect has a different focus. For example, social curiosity (social engagement through the internet, newspapers, and books) and interaction (face-to-face socialization with people) are both part of social relationships, but they have completely different emphases. The previous studies only focused on the part of social relationships or treated all aspects as a whole factor, and only general and vague results were provided regarding which part of the social relationship mediates or moderates the effects of loneliness on depression. Furthermore, it remains unknown whether different aspects of social relationships interact with each other regarding the relationship between loneliness and depression.

In the current study, we set two research questions to address this research gap: (1) Are there any relationships between loneliness, social relationships, and depression symptoms? (2) What role do multifaceted social relationships play in the relationship between loneliness and depression symptoms?

## 2. Materials and Methods

### 2.1. Design

The Community Empowerment and Care for Well-being and Healthy Longevity: Evidence from a Cohort study (CEC) started in 1991 and is ongoing. The survey is conducted every 3 years (the current survey was conducted in 2020), and the self-report questionnaires include sociological, medical, and psychological information. The current study is cross-sectional, with data from the 2017 CEC cohort study.

### 2.2. Participants

In 2017, we surveyed 1116 participants aged ≥65 years. After excluding missing data for six demographic variables, 15 chronic diseases, 18 social relationships, loneliness, and two depression items, 555 participants were finally included in the analysis.

### 2.3. Measures

#### 2.3.1. Loneliness

Loneliness was measured using a signaling question (“Do you experience feelings of loneliness?”), This measurement method has been widely used in studies worldwide as a quick measure of loneliness [43,44,45]. There were four answers to the question: (1) none, (2) rarely, (3) sometimes, and (4) often. Those who answered “non” or “rarely” were considered to have no feelings of loneliness, and those who answered “sometimes” or “often” were considered to have feelings of loneliness.

#### 2.3.2. Depression Symptoms

Depression symptoms were evaluated using a Two-Question Screen. This method has been validated in previous studies worldwide [46,47] and in Japan [48]. The Two-Question Screen consists of (1) During the past month, have you often been bothered by feeling down, depressed, or hopeless? and (2) During the past month, have you often been bothered by little interest in or pleasure in doing things? Each question had two responses: ‘yes’ and ‘no’. Participants who answered ‘Yes’ to either or both questions were considered to have depression symptoms, and those who answered ‘No’ to both questions were considered free of depression symptoms.

#### 2.3.3. Social Relationships

We used the Index of Social Interaction (ISI) scale developed by Tokie Anme and colleagues [32]. This is used to measure participants’ social relationships. The index comprises five aspects of social relationships (independence, social curiosity, interaction, participation, and feeling safe) and 18 items to evaluate social relationships at personal and impersonal levels. Each question has 4 responses in decreasing order of degree or frequency (everyday/always/very, twice a week/sometimes/general, once a week/seldom/little, less than once a month/never/no). If participants respond, “less than once a month” or “never” or “no”, they are given 0 points, while other responses are given 1 point. The total score will be calculated to evaluate the participant’s social relationship level. The total ISI scale was 18, and the scores on the independence, social curiosity, interaction, participation, and feeling safe subscales ranged from 0–4, 0–5, 0–3, 0–4, and 0–2, respectively. It has also been validated among older adults in Japan [49,50,51,52].

The independence subscale comprises four questions: (1) Are you motivated to live a healthy life? (2) Do you have a regular lifestyle? (3) Do you have the motivation to live an active life? (4) Do you talk about an active approach to life?

The social curiosity subscale comprises five questions: (1) Do you read newspapers? (2) Do you read books and magazines? (3) Do you use tools such as the internet, smartphones, and mobile phones? (4) Do you enjoy your hobbies? (5) What do you think you can do to help society?

The interaction subscale includes three questions: (1) How often do you talk to non-family members and non-relatives? (2) How often do you talk to your family and relatives? (3) How often do you visit others?

The participation subscale comprises four questions: (1) How often do you participate in social groups? (2) How well do you know your neighbors? (3) Do you watch television? (4) Do you have a specific role, such as a job or being responsible for household chores?

The feeling safe subscale includes two questions:1) Is there someone you can consult when you are troubled? (2) Is there someone who can help you in an emergency?

#### 2.3.4. Covariables

Age, sex, exercise, alcohol consumption, smoking, economic status, hypertension, stroke, heart disease, diabetes, hyperlipidemia, respiratory disease, stomach disease, kidney disease, muscle disease, cancers, immune diseases, dementia, Parkinson’s disease, eye diseases, and ear diseases were collected using self-report questionnaires and included in the current study as covariables.

Age was divided into 65–74 years and ≥75 years. Sex was classified into two groups: men and women. Exercise was categorized into two groups: exercise and no exercise. Alcohol consumption and smoking were divided into two groups: daily alcohol consumption or smoking and non-daily alcohol consumption or smoking. Economic status was divided into three groups: not good, normal, and good. Finally, sixteen types of diseases were separated into two groups: those with and without diseases.

### 2.4. Statistical Analysis

First, sociodemographic characteristics were described, and the distribution of depressive symptoms was tested using the chi-square test. Subsequently, the intercorrelations of loneliness, depression symptoms, independence subscales, social curiosity subscales, interaction subscales, participation subscales, and feeling safe subscales were tested using Pearson’s correlation analysis.

**Figure 1:**
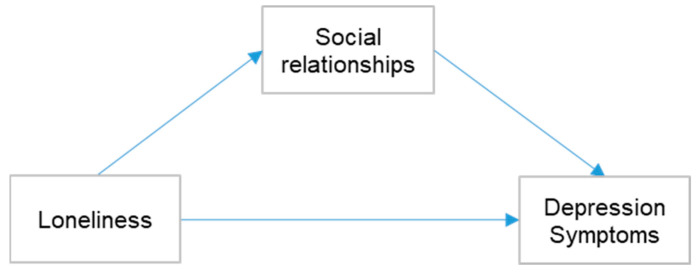
Mediation model with multifaceted social relationships.

Second, we conducted a mediation analysis and set independence, social curiosity, interaction, participation, and feeling safe subscales as mediators to find the potential significant mediation models (Figure 1).

**Figure 2:**
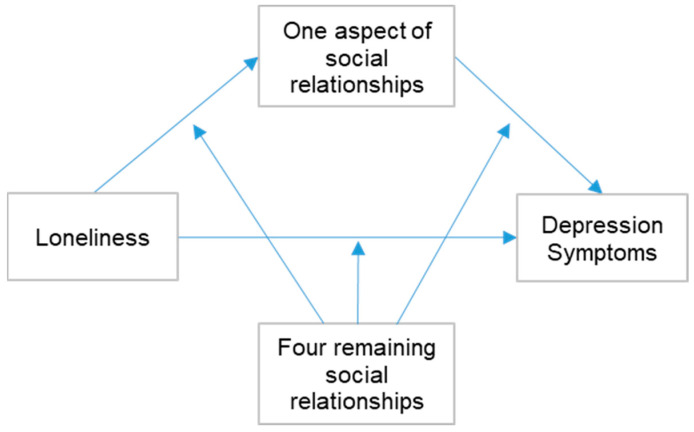
Moderated mediation model with multifaceted social relationships.

Finally, we individually added the other four subscales of social relationship to the significant mediation model to test the moderated mediation effect (Figure 2). α was set to 0.05 in all steps. All analyses were performed using the IBM SPSS 28.0.0 software and the process macro in SPSS (version 28.0.0).

## 3. Results

We described and compared the distribution of demographic characteristics according to depression symptoms (Table 1).

Depressive symptoms were more frequent in those aged 65–74 years than in those aged ≥75 years (32.9% vs. 31.1%), more frequent in men than in women (32.7% vs. 31.7%), more frequent for those undertaking no exercise than in exercise (36.5% vs. 29.2%), more frequent in no daily alcohol consumption and no daily smoking than in daily alcohol consumption and daily smoking (33.8% vs. 26.7% and 33.2% vs. 25.4%), and more frequent in those who did not have a good economic status than in those with normal and good economic status (44.2% vs. 28.5% vs. 18.5%). In addition, there were statistically significant differences in depressive symptoms depending on economic status, heart disease, respiratory disease, kidney disease, cancer, eye disease, and ear diseases.

The intercorrelations among loneliness, depression symptoms, and social relationships are presented in Table 2.

The results showed statistically significant differences between any set of two variables, except loneliness and feeling safe. When we focused on depression symptoms as an outcome variable, the results showed a significant correlation between loneliness and depression symptoms, social curiosity and depression symptoms, independence and depression symptoms, interaction and depression symptoms, participation and depression symptoms, and feeling safe and depressive symptoms.

The mediation effect of the multifaceted social relationship between loneliness and depression symptoms is shown in Table 3.

The direct path of loneliness and depression symptoms was significant in all mediation models (*p* < 0.01). It is noteworthy that social curiosity and participation acted as mediators between loneliness and depression symptoms, with significant indirect paths for loneliness and social curiosity (B = 0.280, SE = 0.101, and *p* < 0.01), social curiosity and depression symptoms (B = 0.317, SE = 0.093, and *p* < 0.01), loneliness and participation (B = 0.195, SE = 0.0070, *p* < 0.01), and participation and depression symptoms (B = 0.367, SE = 0.134, *p* < 0.01).

Social curiosity and participation can be mediators between loneliness and depression symptoms. We then tested whether other aspects of social relationships (independence, interaction, feeling safe) could play the role of moderators (Table 4 and Table 5).

According to Table 4, independence (B = −0.375, SE = 0.167, 95% confidence interval [CI] = −0.703, −0.047) and participation (B = −0.241, SE = 0.110, and 95% CI = −0.458, −0.025) played a moderating role in the pre-indirect path, loneliness—social curiosity. In Table 5, independence (B= −0.256, SE = 0.125, and 95% CI= −0.501, −0.011) played a moderating role in the pre-indirect path of loneliness –participation, and feeling safe (B = 0.515, SE = 0.248, and 95% CI = 0.028, 1.001) played a moderating role in the post-indirect path of participation–depression symptoms. In addition, the results show that interaction can be an individual moderator in the link between loneliness and depression symptoms without any mediator.

Although independence, participation, feeling safe, and interaction may act as moderators, they may have conditional effects. We tested conditional indirect effects and conditional effects of loneliness on depression symptoms at values of multifaceted social relationships (Table 6).

There was a significant effect when the moderator equaled −1 SD independence (B = 0.112, SE = 0.067, 95% CI = 0.015, 0.274) and when the moderator had equal mean independence (B = 0.060, SE = 0.036, 95% CI = 0.003, 0.145). In addition, a significant effect existed when the moderator equaled −1 SD participation (B = 0.114, SE = 0.064, 95% CI = 0.015–0.260). Furthermore, there was a significant effect when the moderator value was −1 SD independence (B = 0.104, SE = 0.068, 95% CI = 0.006, 0.266) with mean independence (B = 0.055, SE = 0.036, 95% CI = 0.003, 0.140). Similarly, a significant effect was observed when feeling safe was the mean value (B = 0.079, SE = 0.045, 95% CI = 0.012, 0.185) with 1 SD (B = 0.090, SE = 0.049, 95% CI = 0.017, 0.204). In addition, the conditional effects of loneliness on depression symptoms moderated by the interaction were significant when the interaction was −1 SD (B = 1.738, SE = 0.314, 95% CI = 1.112, 2.354) with mean (B = 1.283, SE = 0.218, 95% CI = 0.856, 1.710), and 1 SD (B = 1.122, SE = 0.229, 95% CI = 0.673, 1.571).

## 4. Discussion

The ISI measures five aspects of social relationships (independence, social curiosity, interaction, participation, and feeling safe). The current study aimed to determine the role that multifaceted social relationships play in the association between loneliness and depression symptoms. According to the statistical evidence, loneliness affected depressive symptoms through social curiosity and participation. In addition, in the model of loneliness–social curiosity–depression symptoms, independence and participation moderated the effect of loneliness–social curiosity. Moreover, in the model of loneliness–participation–depression symptoms, independence and feeling safe moderated the effect of loneliness–participation and participation–depressive symptoms, respectively.

Although no temporal changes can be derived, we confirmed the results of previous studies, where age and sex did not significantly affect depressive symptoms at older ages [14,15]. Moreover, as in previous studies [17], economic status was significantly associated with depression symptoms. Additionally, we found that the risk of depression symptoms in older adults gradually decreases as their economic status improves. This suggests that economic and material affluence is beneficial in lessening the mental stress of older adults. Therefore, improving the economic status is an effective way of improving quality of life and preventing depression symptoms. Furthermore, in a comparison with previous studies [18,19,20,21,22,23,24,25,26,27], the current study also found that participants with heart, respiratory, kidney, cancer, eye, and ear disorders showed significant differences in depression symptoms compared with those who did not have these diseases. We did not find any statistical evidence for other diseases and exercise affecting depression symptoms. However, this does not mean that there is no association between them. More evidence is required in the future. Future studies are required to explain why the long duration of the disease increases a patient’s stress, whereas these diseases exhibit the same pathological mechanisms as depression symptoms. Therefore, chronic diseases should be investigated further.

As in previous studies [28,29,30,31], loneliness was significantly correlated with depression symptoms. Compared with previous studies [34,35,36,37,38], we found a correlation between various aspects of social relationships (social curiosity, independence, interaction, participation, and feeling safe) and depression symptoms. This may be explained by the fact that satisfying social relationships are essential for good mental and physical health [53,54]. The lack of enriching social relationships causes depression in lonely people.

In the mediation analysis, statistical evidence suggested that social relationships can mediate the relationships between loneliness and depression symptoms. Previous studies showed social resources and participation [40,41] mediates relationships between loneliness and depression symptoms. Besides this evidence, we also found that social curiosity and participation may play a mediating role in this process. A potential mechanism may be that people may be motivated to seek information (from newspapers, books, magazines, and the internet) when they feel lonely [55,56], which, in turn, promotes exploratory acts and affects depression symptoms [57]. Social curiosity and participation as significant mediators between loneliness and depression symptoms should be emphasized in geriatric medicine. Meanwhile, the results of the present study showed that independence, interaction, and feeling safe did not play a mediating role; however, the statistical results suggested a significant direct association with depression symptoms. Thus, a comprehensive improvement in the social relationships of older adults is an effective way to prevent depressive symptoms.

The moderated mediation model found a more complex interaction effect among multifaceted social relationships, loneliness, and depression symptoms, while previous studies only found that social support could moderate the relationships between loneliness and depression symptoms [42]. While social curiosity mediates the effect of loneliness on depression symptoms, independence and participation moderated the effect of loneliness on social curiosity, which changed the whole model’s effect. The same situation occurred in the model with participation as a mediator, independence moderated the effect of loneliness on participation, and feeling safe moderated the effect of participation on depression symptoms. In addition, the interaction could directly moderate the effect of loneliness on depression symptoms. However, social relationships cannot be moderators without limitations in this process. In the model of loneliness–social–curiosity–depression symptoms, independence and participation had a moderating effect only when the value was −1 standard deviation of the mean and when b was −1 standard deviation. Similarly, in the loneliness–participation–depression symptoms model, the moderating effect disappeared when the independence over its mean and feeling safe was lower than its mean. Interaction could act as a moderator in all ranges of values. When preventing depression symptoms in older adults, improving social curiosity and participation while conditionally supporting other social relationships will have a more significant effect.

We performed moderated mediation analysis to test the role of multifaceted social relationships in the association between loneliness and depression symptoms. These results prove the existence of complex interaction effects and provide new insights for the prevention of depression in older adults.

However, our study has some limitations. First, it was a cross-sectional study. We could only identify correlations between social relationships, loneliness, and depression symptoms, but not causal associations. Second, we considered many covariables, which caused a high rate of missing data. Future longitudinal studies with a higher data quality are needed to overcome these limitations.

## 5. Conclusions

The moderated mediation model suggests that social curiosity and participation could mediate the association between loneliness and depression symptoms. In this process, independence, participation, and feeling safe may act as moderators. In the villages in which the study participants live, the government has developed gymnastics and sports meet according to the local culture to increase the socialization of residents. In addition, since many older adults are still working in the Japanese society, this can greatly increase social interaction among older adults and improve their economic status. Moreover, the government provides complimentary medical screening for people over 65 years old, which allows for diseases to be diagnosed and treated earlier. We suggest that other villages increase local cultural activities and provide more employment and medical services and financial support for older adults to prevent depression symptoms in older adults.

## Figures and Tables

**Table 1:** Sociodemographic characteristics and distribution of depressive symptoms (N = 555).

Item	Category	n	%	Depression Symptom					*p*	
				Yes	%	No	%			
Age	65–74	343	61.8	113	32.9	230	67.1		0.657	
	≥75	212	38.2	66	31.1	146	68.9			
Sex	Men	271	48.8	86	31.7	185	68.3		0.799	
	Women	284	51.2	93	32.7	191	67.3			
Exercise	Yes	325	58.6	95	29.2	230	70.8		0.070	
	No	230	41.4	84	36.5	146	63.5			
Alcohol consumption	Daily	120	21.6	32	26.7	88	71.3		0.139	
	Non-daily	435	78.4	147	33.8	288	66.2			
Smoking	Daily	67	12.1	17	25.4	50	74.6		0.199	
	Non-daily	488	87.9	162	33.2	326	66.8			
Economic	Not good	129	23.2	57	44.2	72	55.8		0.004	
	Normal	382	68.8	109	28.5	273	71.5			
	Good	44	7.9	13	18.5	31	81.5			
Hypertension	Yes	247	44.5	75	30.4	172	69.6		0.394	
	No	308	55.5	104	33.8	204	66.2			
Stroke	Yes	29	5.2	9	31.0	20	69.0		0.885	
	No	526	94.8	170	32.3	356	67.7			
Heart Disease	Yes	46	8.3	21	45.7	25	54.3		0.042	
	No	509	91.7	158	31.0	351	69.0			
Diabetes	Yes	86	15.5	28	32.6	58	67.4		0.947	
	No	469	84.5	151	32.2	318	67.8			
Hyperlipidemia	Yes	65	11.7	23	35.4	42	64.6		0.565	
	No	490	88.3	156	31.8	334	68.2			
Respiratory disease	Yes	27	49	14	51.9	13	48.1	0.026		
	No	528	95.1	165	31.3	363	68.7			
Stomach disease	Yes	19	3.4	10	52.6	9	47.4	0.053		
	No	536	96.6	169	31.5	367	68.5			
Kidney disease	Yes	40	7.2	22	55.0	18	45.0	0.001		
	No	515	92.8	157	30.5	358	69.5			
Muscle disease	Yes	58	10.5	24	41.4	34	58.6	0.116		
	No	497	89.5	155	31.2	342	68.8			
Cancers	Yes	21	3.8	11	52.4	10	47.6	0.044		
	No	534	96.2	169	31.6	366	68.4			
Immune diseases	Yes	9	1.6	2	22.2	7	77.8	0.516		
	No	546	98.4	177	32.4	369	67.6			
Dementia	Yes	11	2	6	51.5	5	48.5	0.110		
	No	544	98	173	31.8	371	68.2			
Parkinson’s disease	Yes	4	0.7	3	75.0	1	25.0	0.066		
	No	511	99.3	176	34.4	375	65.6			
Eye diseases	Yes	126	22.7	55	43.7	71	56.3	0.002		
	No	429	77.3	124	28.9	305	71.1			
Ear diseases	Yes	35	6.3	22	62.9	13	37.1	<0.001		
	No	520	93.7	157	30.2	363	69.8			

**Table 2:** Intercorrelations among loneliness, depression symptoms, and social relationships (N = 555).

	a	b	c	d	e	f
Loneliness	1					
Depression symptoms	0.296 **	1				
Social curiosity	0.244 **	0.195 **	1			
Independence	0.138 **	0.121 **	0.556 **	1		
Interaction	0.104 *	0.130 **	0.360 **	0.329 **	1	
Participation	0.179 **	0.177 **	0.469 **	0.434 **	0.406 **	1
Feeling safe	0.062	0.111 **	0.110 **	0.153 **	0.311 **	0.197 **

* *p* < 0.05; ** *p* < 0.01.

**Table 3:** Mediation analysis.

Outcome	Variable	B	SE
Social curiosity	Loneliness	0.280 **	0.101
Depression symptom	Social curiosity	0.317 **	0.093
Depression symptom	Loneliness	1.235 **	0.218
R2	0.303		
F	10.504		
Independence	Loneliness	0.080	0.045
Depression symptom	Independence	0.414 *	0.200
Depression symptom	Loneliness	1.268 **	0.216
R2	0.145		
F	4.107		
Interaction	Loneliness	0.055	0.043
Depression symptom	Interaction	0.439 *	0.212
Depression symptom	Loneliness	1.285 **	0.217
R2	0.059		
F	1.526		
Participation	Loneliness	0.195 **	0.070
Depression symptom	Participation	0.367 **	0.134
Depression symptom	Loneliness	1.242 **	0.218
R2	0.153		
F	4.382		
Feeling safe	Loneliness	0.063	0.037
Depression symptom	Feeling safe	0.396	0.245
Depression symptom	Loneliness	1.273	0.216
R2	0.063		
F	1.624		

* *p* < 0.05; ** *p* < 0.01.

**Table 4:** Moderated mediation analysis (social curiosity, independence, interaction, participation, and feeling safe as moderators).

Outcome	Variable	B	SE	*t*/*z*	LLCI	ULCI
Social curiosity	Loneliness	0.191 *	0.088	2.170	0.018	0.363
	Independence	1.235 **	0.103	11.959	1.033	1.438
	Loneliness × Independence	−0.375 *	0.167	−2.243	−0.703	−0.047
Depression symptom	Loneliness	1.239 **	0.220	5.616	0.807	1.672
	Social curiosity	0.287 **	0.109	2.647	0.075	0.450
	Independence	0.008	0.610	0.013	−1.187	1.203
	Loneliness × Independence	−0.680	0.497	−1.369	−1.654	0.294
	Social curiosity × Independence	−1.114	0.187	−0.609	−0.479	0.252
Social curiosity	Loneliness	0.237 *	0.095	2.487	0.050	0.424
	Interaction	0.674 **	0.138	4.897	0.404	0.945
	Loneliness × Interaction	0.233	0.193	1.207	−0.146	0.612
Depression symptom	Loneliness	1.242 **	0.221	5.607	0.808	1.676
	Social curiosity	0.306 **	0.104	2.933	0.101	0.511
	Interaction	0.770 *	0.381	2.020	0.023	1.516
	Loneliness × Interaction	−1.161 *	0.502	−2.314	−2.144	−0.176
	Social curiosity × Interaction	−0.050	0.148	−0.336	−0.341	0.241
Social curiosity	Loneliness	0.165	0.093	1.770	−0.018	0.348
	Participation	0.680 **	0.077	8.781	0.528	0.832
	Loneliness × Participation	−0.241 *	0.110	−2.187	−0.458	−0.025
Depression symptom	Loneliness	1.204 **	0.220	5.466	0.772	1.635
	Social curiosity	0.254 *	0.107	2.374	0.044	0.463
	Participation	0.363	0.218	1.665	−0.064	0.791
	Loneliness × Participation	−0.290	0.272	−1.067	−0.823	0.243
	Social curiosity × Participation	0.006	0.080	0.071	−0.150	0.161
Social curiosity	Loneliness	0.259 **	0.100	2.587	0.062	0.456
	Feeling safe	0.150	0.166	0.904	−0.176	0.472
	Loneliness × Feeling safe	0.420	0.231	1.819	−0.334	0.873
Depression symptom	Loneliness	1.194 **	0.220	5.437	0.764	1.625
	Social curiosity	0.323 **	0.096	3.372	0.135	0.511
	Feeling safe	0.478	0.361	1.327	−0.228	1.185
	Loneliness × Feeling safe	−0.201	0.496	−0.405	−1.174	0.772
	Social curiosity × Feeling safe	0.231	0.181	1.278	−0.123	0.586

* *p* < 0.05; ** *p* < 0.01.

**Table 5:** Moderated mediation analysis (social curiosity, interaction, participation, and feeling safe as moderators).

Outcome	Variable	B	SE	*t*/*z*	LLCI	ULCI
Participation	Loneliness	0.117	0.065	1.800	−0.011	0.245
	Social curiosity	0.300 **	0.035	8.641	0.232	0.368
	Loneliness × Social curiosity	−0.064	0.050	−1.282	−0.162	0.034
Depression symptom	Loneliness	1.208 **	0.220	5.484	0.776	1.640
	Participation	0.224	0.165	1.355	−0.100	0.548
	Social curiosity	0.313 *	0.130	2.409	0.058	0.568
	Loneliness × Social curiosity	−0.145	0.174	0.834	−0.486	0.196
	Participation × Social curiosity	0.001	0.079	0.013	−0.154	0.156
Participation	Loneliness	0.151 *	0.066	2.292	0.022	0.280
	Independence	0.643 **	0.077	8.329	0.492	0.795
	Loneliness × Independence	−0.256 *	0.125	−2.049	−0.501	−0.011
Depression symptom	Loneliness	1.245 **	0.221	5.648	0.813	1.677
	Participation	0.297 *	0.146	2.031	0.010	0.584
	Independence	0.484	0.368	1.316	−0.237	1.204
	Loneliness × Independence	−0.808	0.477	−1.695	−1.743	0.127
	Participation × Independence	−0.029	0.197	−0.146	−0.415	0.358
Participation	Loneliness	0.161 *	0.065	2.466	0.033	0.289
	Interaction	0.652 **	0.094	6.926	0.467	0.836
	Loneliness × Interaction	−0.053	0.132	−0.402	−0.312	0.206
Depression symptom	Loneliness	1.238 **	0.220	5.631	0.807	1.670
	Participation	0.323 *	0.153	2.115	0.024	0.623
	Interaction	0.798 *	0.385	2.073	0.044	1.553
	Loneliness × Interaction	−0.949 *	0.479	−1.981	−1.888	−0.010
	Participation × Interaction	0.055	0.188	0.291	−0.314	0.423
Participation	Loneliness	0.173 *	0.069	2.515	0.038	0.309
	Feeling safe	0.238	0.114	2.081	0.013	0.462
	Loneliness × Feeling safe	0.265	0.159	1.666	−0.047	0.577
Depression symptom	Loneliness	1.193 **	0.219	5.436	0.763	1.623
	Participation	0.405 **	0.142	2.854	0.127	0.684
	Feeling safe	0.570	0.377	1.513	−0.168	1.308
	Loneliness × Feeling safe	−0.095	0.510	−0.185	−1.094	0.905
	Participation × Feeling safe	0.515 *	0.248	2.074	0.028	1.001

* *p* < 0.05; ** *p* < 0.01.

**Table 6:** Conditional indirect effects and conditional effects of loneliness on depression symptoms at values of multifaceted social relationships.

Mediator	Moderator	B	SE	LLCI	ULCI
Social curiosity	−1 SD independence	0.122	0.067	0.015	0.274
	Mean independence	0.060	0.036	0.003	0.145
	1 SD independence	0.043	0.034	−0.015	0.121
Social curiosity	−1 SD participation	0.114	0.064	0.015	0.260
	Mean participation	0.052	0.036	−0.007	0.133
	1 SD participation	0.005	0.038	−0.075	0.076
Participation	−1 SD independence	0.104	0.068	0.006	0.266
	Mean independence	0.055	0.036	0.003	0.140
	1 SD independence	0.042	0.032	−0.008	0.118
Participation	−1 SD feeling safe	0.038	0.043	−0.037	0.136
	Mean feeling safe	0.079	0.045	0.012	0.185
	1 SD feeling safe	0.09	0.049	0.017	0.204
No mediator	−1 SD interaction	1.738	0.314	1.112	2.354
	Mean interaction	1.283	0.218	0.856	1.710
	1 SD interaction	1.122	0.229	0.673	1.571

## Data Availability

Not applicable.
